# Real-World Time on Treatment with First-Line Pembrolizumab Monotherapy for Advanced NSCLC with PD-L1 Expression ≥ 50%: 3-Year Follow-Up Data

**DOI:** 10.3390/cancers14041041

**Published:** 2022-02-18

**Authors:** Vamsidhar Velcheti, Xiaohan Hu, Yeran Li, Hazem El-Osta, M. Catherine Pietanza, Thomas Burke

**Affiliations:** 1 Perlmutter Cancer Center, NYU Langone, New York, NY 10016, USA; 2 Merck & Co., Inc., Kenilworth, NJ 07033, USA; xiaohan.hu@merck.com (X.H.); yeran.li@merck.com (Y.L.); hazem.el-osta@merck.com (H.E.-O.); cathy.pietanza@merck.com (M.C.P.); thomas_burke2@merck.com (T.B.)

**Keywords:** advanced NSCLC, *KRAS* mutation, lines of therapy, overall survival, pembrolizumab, treatment duration

## Abstract

**Simple Summary:**

Lung cancer is the leading cause of cancer-related death in the United States (US), and real-world studies are needed to understand effectiveness of cancer therapies for patients treated outside of cancer clinical trials. Pembrolizumab, an immunotherapy agent that aids the body’s immune system in fighting cancer, is administered for up to 2 years when treating advanced non-small cell lung cancer (NSCLC). We evaluated the real-world time on treatment (rwToT), a surrogate indicator that has been associated with survival in NSCLC studies, for over 1000 patients with advanced NSCLC treated initially with pembrolizumab at US oncology clinics. The median rwToT for patients with good performance status (similar to those in clinical trials) was 7.4 months, consistent with the median treatment duration in the KEYNOTE-024 trial (7.9 months). Our findings suggest long-term benefit of first-line pembrolizumab for patients with advanced NSCLC and good performance status at the start of therapy who are treated in real-world settings.

**Abstract:**

Our aim was to evaluate real-world time on treatment (rwToT), overall and by *KRAS* mutation status, with first-line pembrolizumab monotherapy for advanced non-small cell lung cancer (NSCLC) in real-world oncology practice in the US. rwToT is a readily available, intermediate-range endpoint that is moderately to highly correlated with overall survival in clinical trials and real-world data. Using deidentified electronic medical record data, we studied patients with ECOG performance status (PS) of 0–2 who initiated pembrolizumab (1 November 2016 to 31 March 2020) for advanced NSCLC with programmed death-ligand 1 (PD-L1) expression ≥ 50% and without *EGFR/ALK/ROS1* genomic alterations. The data cutoff was 31 March 2021, and the median study follow-up was 34 months. The Kaplan–Meier median rwToT with first-line pembrolizumab monotherapy was 7.4 months (95% CI, 6.3–8.1) for 807 patients with PS 0–1, which was consistent with the median treatment duration in the KEYNOTE-024 trial (7.9 months). The median rwToT for 237 patients with PS 2 was 2.1 months (95% CI, 1.4–2.8). For those with *KRAS*-mutated and *KRAS* wild-type nonsquamous NSCLC and PS 0–1, the median rwToT was 7.6 months and 7.0 months, respectively. Our findings suggest long-term benefit of first-line pembrolizumab monotherapy for advanced NSCLC with PD-L1 expression ≥ 50% in real-world settings in the US, particularly for patients with good performance status at the start of therapy, irrespective of *KRAS* status.

## 1. Introduction

Lung cancer is the third most common cancer in the United States (US), often presenting at late stages. While it remains the leading cause of cancer-related deaths in both men and women [1], declines in mortality from lung cancer have been recorded in the past decade [2,3]. Recent accelerated declines in mortality from non-small cell lung cancer (NSCLC), the most common subtype, are attributed to both declining incidence (because of decreased smoking rates in the US), as well as expanding treatment options [2]. Newer treatment options include therapies targeted against oncogenic drivers, such as *EGFR* mutations and *ALK*, *RET*, and *ROS1* rearrangements, and, since 2015, immune checkpoint inhibitors (ICI) targeting the programmed death 1 (PD-1) pathway, such as the anti-PD-1 antibodies nivolumab, pembrolizumab, and cemiplimab and the anti-PD-ligand 1 (PD-L1) antibodies atezolizumab and durvalumab.

The choice of first-line systemic anticancer therapy for unresectable advanced NSCLC is guided by tumor histology, biomarkers (e.g., tumor PD-L1 expression), and whether targetable genomic alterations are present, in addition to patient clinical status and preferences [4,5,6]. For patients who have unresectable advanced or metastatic NSCLC with PD-L1 expression ≥ 50% and no targetable genomic alterations, the preferred first-line therapies listed in US National Comprehensive Cancer Network (NCCN) guidelines include pembrolizumab, atezolizumab, or cemiplimab-rwlc as monotherapy or pembrolizumab-combination therapy, the latter chosen according to NSCLC histopathology [7]. Continued (maintenance) therapy, chosen according to initial ICI therapy, is then recommended until disease progression or unacceptable toxicity (or for up to 2 years in the case of pembrolizumab) [7].

Testing for targetable genomic alterations (e.g., *EGFR/ALK/ROS1*) is recommended before initiating first-line therapy, with the appropriate targeted therapy then administered for those tumors with positive results. *KRAS* mutations are the most common mutations in lung adenocarcinomas in the US, occurring in approximately 25% of cases [8]. Sotorasib is a KRAS^G12C^ inhibitor that was recently approved in the US and by the European Commission for treating *KRAS*-mutated NSCLC that has progressed after at least one prior line of systemic therapy, but there is currently no therapeutic approach specifically targeting *KRAS* mutations in the first-line setting. However, recent evidence suggests that the activity of first-line pembrolizumab and other ICIs appears to be retained regardless of *KRAS* mutation status [4,9,10,11].

In the KEYNOTE-024 (KN024) clinical trial, pembrolizumab monotherapy for previously untreated metastatic NSCLC (with PD-L1 tumor proportion score (TPS) ≥ 50%, *EGFR*/*ALK*-negative) was continued for 35 cycles (~2 years) or until a patient experienced disease progression or treatment-related adverse events of unacceptable severity, they withdrew consent, or the investigator decided to withdraw the patient (whichever came first) [12,13,14]. With 5 years of follow-up in KN024, the median duration of pembrolizumab therapy was 7.9 months (range, 1 day to 30.2 months) and the Kaplan–Meier estimated 5-year overall survival (OS) rate was 32% in the pembrolizumab arm; 39/151 patients (26%) completed 35 cycles of pembrolizumab, of whom 82% were alive at 5 years [14].

Controlled clinical trials such as KN024 are designed to maximize internal validity; however, the importance of also understanding treatment effectiveness in real-world settings (generalizability) is now well-recognized [15,16]. Ideal real-world studies include large patient cohorts and long-term follow-up, both challenging criteria in light of how recently first-line ICI therapies have been deployed for treating advanced NSCLC. Moreover, reliable and measurable endpoints are needed in observational studies. The determination of real-world time on treatment (rwToT), also known as time-to-treatment discontinuation (TTD), has been studied as an intermediate-range endpoint that is readily assessable in many electronic health record (EHR) and claims databases. For continuously administered therapies, such as ICIs, rwToT is highly correlated at the patient-level with progression-free survival (PFS) and moderately to highly correlated with OS in NSCLC clinical trials [17,18], as well as being moderately to highly correlated with OS in diverse real-world datasets [19,20,21].

In our prior observational study of patients with metastatic (stage IV) NSCLC, with PD-L1 expression ≥ 50%, treated with first-line pembrolizumab monotherapy at US oncology practices, the median rwToT was 6.9 months (95% CI, 5.6–8.3) for the 386 patients with good performance status; however, the follow-up time was insufficient to assess the percentage of patients remaining on therapy at 24 months [22]. The aim of the present study was to further evaluate rwToT with first-line pembrolizumab monotherapy over a longer follow-up period for an expanded patient population treated at US oncology clinics, including those with unresectable stage-IIIB/C NSCLC. A secondary objective was to evaluate rwToT according to *KRAS* mutation status.

## 2. Materials and Methods

### 2.1. Patients and Data Source

This retrospective observational study utilized deidentified EHR data from the US nationwide Flatiron Health advanced NSCLC database, which includes patients with pathologically confirmed advanced NSCLC (newly diagnosed or recurrent/progressive disease) who had at least two visits recorded in the database on or after 1 January 2011 [23]. We studied patients who were at least 18 years old at the time of initiating first-line pembrolizumab from 1 November 2016 through 31 March 2020 for treating unresectable NSCLC (stage IIIB/C or stage IV) with PD-L1 expression ≥ 50%. Other inclusion criteria were no known *EGFR*, *ALK*, or *ROS1* genomic alterations and, for nonsquamous tumors, documented wild-type *EGFR* and no *ALK* gene rearrangements.

We restricted the analyses to patients with Eastern Cooperative Oncology Group performance status (ECOG PS) of 0–2; patients with unknown ECOG PS and those with ECOG PS of 3–4 were excluded. Patients participating in a clinical trial were also excluded, as were those with no recorded activity in the database within 90 days (inclusive) of the advanced NSCLC diagnosis. The data cutoff was on 31 March 2021, thereby enabling at least 12 months of potential patient follow-up after first-line pembrolizumab initiation.

At the time of this study, the Flatiron Health database included longitudinal, deidentified patient data sourced from EHRs of patients treated at approximately 800 sites, including both community and academic oncology clinics [23]. The use of the Flatiron Health database for characterizing patient populations and determining real-world survival and treatment-based endpoints has been described in detail in prior publications [20,24,25]. Ethical approval of the study protocol (number RWE-001, titled The Flatiron Health Real World Evidence Parent Protocol), including a waiver of informed consent, was obtained before conducting the study from the WCG Institutional Review Board (Protocol approval ID: IRB00000533; tracking number: 420180044). The IRB approval is updated periodically, with the latest approval dated 29 November 2021. Flatiron Health, Inc. did not participate in the analysis of the data.

### 2.2. Assessments and Statistical Analyses

We used descriptive statistics to summarize, according to ECOG PS (PS 0–1 and 2), patient demographic and clinical characteristics, the number of pembrolizumab cycles administered, and the percentage of patients initiating each category of systemic anticancer regimen in subsequent treatment lines. Test results for PD-L1 expression and tumor genomic alterations were abstracted from unstructured information in EHRs, pathology reports, or clinical notes; the Charlson comorbidity index score was derived from listed comorbidities [26]. Lines of therapy were identified using Flatiron Health oncologist-defined business rules [20]. For each subsequent line of therapy, mutually exclusive regimen classes were assigned in hierarchical order (anti-PD-1/PD-L1-based therapy > anti-vascular endothelial growth factor (VEGF)-based therapy > platinum-based chemotherapy combinations > nonplatinum-based chemotherapy combinations > single-agent chemotherapy > other therapy).

The Kaplan–Meier method was used to estimate rwToT with pembrolizumab, as previously described [22,27]. In brief, rwToT was determined as [(date of last recorded pembrolizumab dose − date of first recorded dose) + 1 day], defining treatment discontinuation at the last noncancelled order or administration date if patients died during pembrolizumab therapy or initiated a next line of therapy, or if there was a gap of ≥120 days between the last recorded pembrolizumab dose and last clinical contact date in the dataset [22]. We determined the median rwToT and the restricted mean rwToT, as well as the percentage of patients still receiving pembrolizumab at 12, 24, 36, and 48 months [22], stratified by ECOG PS. The restricted mean was defined as the mean calculated with the assumption of a maximum pembrolizumab exposure at designated time points (i.e., 12, 24, 36, and 48 months in this study) for those patients still on treatment at data cutoff. For timepoints at which the data were considered not sufficiently mature, the restricted mean rwToT was extrapolated using parametric functions fitted to the Kaplan–Meier data [22,28,29].

A subanalysis was conducted for patients with ECOG PS 0–1 to evaluate the rwToT according to the *KRAS* mutation status of nonsquamous tumors (overall, positive, wild-type, unknown). In addition, as a sensitivity analysis, we determined the rwToT for patients with stage-IV NSCLC at diagnosis, stratified by ECOG PS (PS 0–1 and 2). No statistical analyses were conducted for between-group comparisons.

Statistical analyses were conducted using SAS software, version 9.4 (SAS Institute, Cary, NC, USA).

## 3. Results

### 3.1. Patient Characteristics

**Figure 1:**
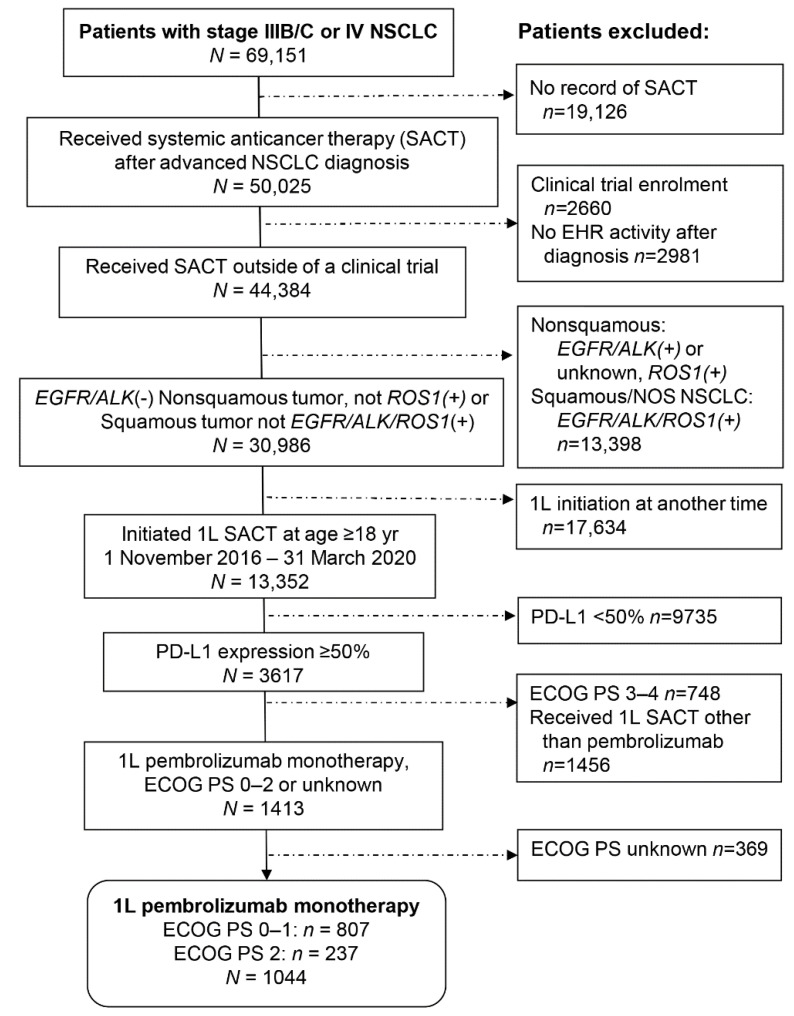
Selection of eligible patients from the Flatiron Health Advanced NSCLC Database. 1L: first-line; ECOG PS: Eastern Cooperative Oncology Group performance status; EHR: electronic health record; NSCLC: non-small cell lung cancer; PD-L1: programmed death-ligand 1; SACT: systemic anticancer therapy.

We identified 1044 patients with ECOG PS of 0–2 who were treated with first-line pembrolizumab monotherapy for advanced NSCLC with PD-L1 expression ≥ 50% and no recorded *EGFR/ALK/ROS1* genomic alteration, including 807 patients (77%) with ECOG PS of 0–1 and 237 (23%) with ECOG PS of 2 (Figure 1).

The median patient age was 72 years in the PS 0–1 cohort and 75 years in the PS 2 cohort, including 40% and 51% of patients ≥ 75 years old, respectively (Table 1). Half of the patients in each cohort were men. Approximately three-quarters of those with known race in each cohort were White, and 9% and 11% were Black, respectively. Almost all patients (98%) were treated at community oncology clinics (Table S1).

The percentages of patients with nonsquamous tumors were 67% and 72% in PS 0–1 and PS 2 cohorts, respectively. Of the nonsquamous tumors, 30% and 29%, respectively, were positive for *KRAS* mutations, representing one-half of tumors with known results in each cohort (Table 1).

### 3.2. First-Line Treatment with Pembrolizumab Monotherapy: Real-World Time on Treatment

The median time from first-line pembrolizumab initiation to data cutoff was 34.1 and 33.5 months in the PS 0–1 and PS 2 cohorts, respectively; individual patients were followed for a median of 17.4 and 5.7 months, respectively (Table 2).

**Figure 2:**
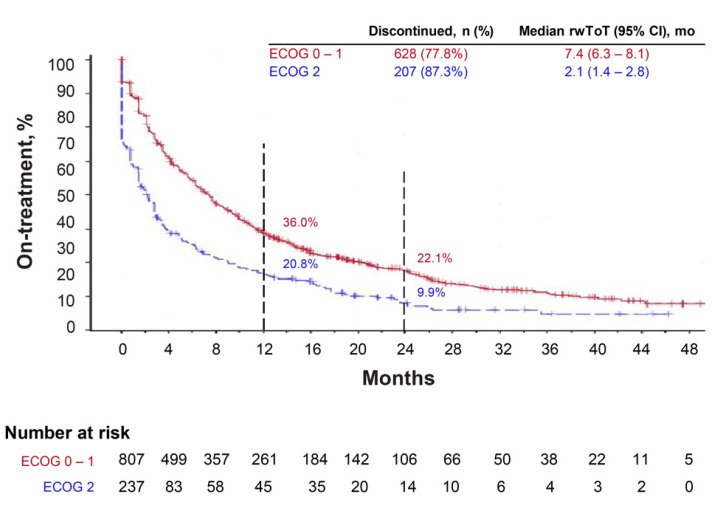
Kaplan–Meier plot depicting real-world time on treatment (rwToT) with first-line pembrolizumab monotherapy for patients with advanced NSCLC, according to ECOG performance status.

In the PS 0–1 cohort, the median rwToT with pembrolizumab was 7.4 months (95% CI, 6.3–8.1), and 22.1% of patients (95% CI, 19.1–25.3) remained on pembrolizumab at 24 months; the restricted mean rwToT at 24 months was 10.3 months (95% CI, 9.7–11.0; Table 2, Figure 2). In the PS 2 cohort, the median rwToT with pembrolizumab was 2.1 months (95% CI, 1.4–2.8), and 9.9% of patients (95% CI, 6.1–14.6) remained on pembrolizumab at 24 months; the restricted mean rwToT at 24 months was 5.9 months (95% CI, 4.9–7.0 (Lognormal)).

Among patients with at least 2 years of theoretical follow-up, the median number of pembrolizumab cycles administered was 10.0 and 3.5 in the PS 0–1 and PS 2 cohorts, respectively (Table S2). In the PS 0–1 cohort, 378 patients (64%) received six or more cycles, and in the PS 2 cohort, 66 (38%) patients received ≥ 6 cycles. A total of 92 (16%) and 14 (8%) received ≥ 35 cycles, respectively. Most patients were treated with the 200-mg dose of pembrolizumab, approved by the US FDA for administration every 3 weeks; 16 (3%) and 2 (1%) patients, respectively, received the 400-mg dose, approved on 28 April 2020 for administration every 6 weeks.

### 3.3. Subanalysis of rwToT by KRAS Mutation Status for Nonsquamous NSCLC, PS 0–1

**Figure 3:**
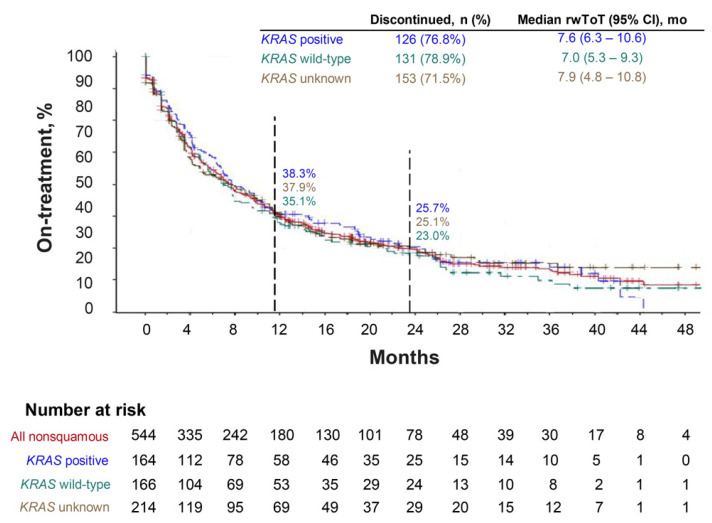
Kaplan–Meier plot depicting real-world time on treatment (rwToT) with pembrolizumab by *KRAS* mutation status for patients with ECOG performance status 0–1.

Of the 544 patients with nonsquamous NSCLC who had good performance status (PS 0–1), the *KRAS* mutation status was available for 330 patients, including 164 (50%) with *KRAS*-mutated NSCLC and 166 (50%) with *KRAS* wild-type NSCLC. The median rwToT with first-line pembrolizumab monotherapy was 7.6 months (95% CI, 6.3–10.6) for patients with *KRAS*-mutated NSCLC, 7.0 months (95% CI, 5.3–9.3) for those with *KRAS* wild-type tumors, and 7.6 months (95% CI, 6.3–8.8) for all patients with nonsquamous tumors (Table 3, Figure 3). The 24-month restricted mean rwToT was 11.3 and 10.3 months in the *KRAS*-mutated and wild-type cohorts, respectively, and the Kaplan–Meier estimates of percentages of patients still on treatment at 24 months were 25.7% (95% CI, 18.8–33.1%) and 23.0% (95% CI, 16.4–30.3%), respectively (Table 3).

### 3.4. Sensitivity Analysis Including Only Patients with Stage-IV NSCLC at Diagnosis

The initial NSCLC diagnosis was made at stage IV for most patients, including 566 (70%) in the PS 0–1 cohort and 178 (75%) in the PS 2 cohort. The baseline characteristics of these patients resembled those of the full cohorts, including median ages of 71 years and 75 years, respectively, and approximately 50% men, as summarized in Table S3.

The results of the rwToT analyses showed a similar pattern to those of the full cohorts: the median rwToT for patients with PS 0–1 was 6.5 months (95% CI, 5.6–7.6), and for those with PS 2, it was 1.6 months (95% CI, 0.7–2.8; Table S4, Figure S1). An estimated 21.2% (95% CI, 17.7–25.0) and 7.7% (95% CI, 4.0–13.0) of patients with PS 0–1 and PS 2, respectively, remained on pembrolizumab therapy at 24 months.

### 3.5. Subsequent Systemic Anticancer Therapy

In the PS 0–1 cohort, 263 patients (33%) received second-line systemic anticancer therapy, most commonly anti-PD-1/PD-L1-based therapy (105, 40%) or platinum-based chemotherapy (88, 33%; Table 4). Second-line pembrolizumab was administered as monotherapy to 25 patients and in combination with other agents to 66 patients, most commonly with carboplatin and pemetrexed (details are in Table S5). Third-line systemic therapy was administered to 91 of the 263 patients who received second-line therapy (35%), most commonly as anti-PD-1/PD-L1-based therapy (34, 37%) or single-agent chemotherapy (24, 26%).

In the PS 2 cohort, 39 patients (16%) continued to second-line therapy, 17 (44%) of whom received platinum-based chemotherapy, and 11 (28%) of whom received anti-PD-1/PD-L1-based therapy (Table 4). Of the latter 11 patients, 10 received pembrolizumab, 3 as monotherapy and 7 in combination with other agent(s) (Table S5). Ten patients (10/39, 26%) continued to third-line therapy, most commonly anti-PD-1/PD-L1-based therapy (4, 40%) or single-agent chemotherapy (3, 30%).

## 4. Discussion

In this retrospective observational study, the median rwToT with first-line pembrolizumab monotherapy was 7.4 months for the 807 patients in the PS 0–1 cohort and 2.1 months for the 237 patients in the PS 2 cohort. These patients, with advanced NSCLC, PD-L1 expression ≥ 50%, and no documented *EGFR/ALK/ROS1* genomic alterations, were treated with first-line pembrolizumab monotherapy in the real-world setting of US oncology practices. With a median follow-up of almost 3 years (34 months) in both the PS 0–1 and PS 2 cohorts, the Kaplan–Meier estimated percentages of patients still on pembrolizumab treatment at 24 months were 22% and 10%, respectively. Among patients in the PS 0–1 cohort, the median rwToT was 7.6 months for those with *KRAS*-positive NSCLC and 7.0 months for those with *KRAS* wild-type NSCLC, both resembling that of the full PS 0–1 cohort with nonsquamous NSCLC (median 7.6 months).

The median rwToT of 7.4 months for all patients with ECOG PS 0–1 was consistent with the analogous endpoint in KN024 (median pembrolizumab duration, 7.9 months) and the subpopulation with stage-IV NSCLC, with PD-L1 ≥ 50%, in KN042 (median, 6.6 months), as calculated in our prior study [13,22,30]. Moreover, the 24-month restricted mean rwToT was 10.3 months (95% CI, 9.7–11.0), also consistent with the 24-month restricted mean durations of pembrolizumab treatment in KN024 and the KN042 subpopulation of 11.0 months (95% CI, 9.5–12.5) and 10.4 months (95% CI, 9.3–11.5), respectively [13,22,30].

Among patients with at least 2 years of theoretical follow-up (i.e., from pembrolizumab initiation until data cutoff), 16% of patients with ECOG PS 0–1 had received ≥ 35 pembrolizumab cycles. By contrast, in KN024, 26% of patients had completed 35 cycles at the time of the most recent (5-year) assessment. This difference could be attributable to differences in follow-up length of the present study relative to KN024 (median, 34 vs. 60 months, respectively) [14], as well as potential treatment interruptions, which are common in routine clinical practice, and administration of the 400-mg dose (*n* = 16) after FDA approval in 2020. Of interest, at data cutoff, only one-third of patients with ECOG PS 0–1 (33%) had continued to a subsequent systemic therapy, and 40% of these patients received second-line anti-PD-1/PD-L1-based therapy.

We observed that patients were somewhat older in the PS 0–1 cohort of the present study than in the pembrolizumab arms of the KEYNOTE trials (median age 72 vs. 65 and 63 in KN024 and KN042) and included proportionately more women (49% vs. 40% and 32% in KN024 and KN042) [13,22,30]. These differences between real-world and cancer trial patient populations in terms of age and sex distribution are commonly reported [27,31,32,33].

In the sensitivity analysis of results for the patients in our study with stage-IV NSCLC at diagnosis, the median rwToT of 6.5 months and 1.6 months in PS 0–1 and PS 2 cohorts, respectively, were shorter than those for all patients, likely reflective of more severe disease with an initial diagnosis at stage IV. The median rwToT in our prior similarly designed, retrospective study was 6.9 months for patients with stage-IV NSCLC in the PS 0–1 cohort [22]. With the longer follow-up in the present study relative to that study, the 24-month restricted mean rwToT values were similar (10.3 months; 95% CI, 9.7–11.0 vs. 10.5 months; 9.4–11.7 (Weibull) in the prior study), and this was also the case for patients included in stage-IV sensitivity analyses (10.0 months; 95% CI, 9.2–10.7) [22].

Other recently published studies have used the Flatiron Health database to evaluate outcomes with first-line ICI therapy for broadly defined patient populations with advanced/metastatic NSCLC [34,35]. For 810 patients with stage-IV NSCLC at diagnosis (no *EGFR/ALK* genomic alteration), the median rwToT was 4.7 months (95% CI, 4.2–5.8) months [34]. That study included all patients irrespective of PD-L1 expression and performance status (68% had PD-L1 ≥ 50%, ~50% had ECOG PS of 0–1, and 25% had unknown ECOG PS), and the 6-month minimum follow-up may have limited the ability to capture the full treatment course. Similarly, using the Flatiron Health database, with a median follow-up of approximately 7 months, Waterhouse et al. [35] estimated median rwToT with first-line ICI monotherapy of 4.3 and 4.4 months for squamous and nonsquamous advanced NSCLC, respectively (70% of 3041 patients with PD-L1 ≥ 50%; 53% with ECOG PS of 0–1).

At the time of this writing, we were unable to find rwToT information specifically for first-line atezolizumab or cemiplimab, perhaps because of their later first-line approvals (2020 and 2021, respectively, in the US). In key first-line clinical trials, the median duration of atezolizumab monotherapy was 5.3 months, and the median duration of cemiplimab monotherapy was 6.3 months [36,37].

In the present study, half of those tested with nonsquamous tumors had *KRAS*-mutated NSCLC, which was not unexpected because *KRAS* mutations are more prevalent in PD-L1-expressing tumors. Similarly, in another study restricted to high PD-L1-expressing tumors (PD-L1 ≥ 50%) and no *EGFR/ALK/ROS1* genomic alteration, Noordhof et al. [11] found that 57% of metastatic lung adenocarcinomas carried *KRAS* mutations. Instead, in the broader sample not restricted by PD-L1 expression included in the Lung Cancer Mutation Consortium study, 27% of 1655 patients had metastatic lung adenocarcinomas harboring a *KRAS* mutation [8]. We found that the median and restricted mean rwToT with pembrolizumab monotherapy were similar for *KRAS*-mutated vs. wild-type vs. all nonsquamous tumors, with Kaplan–Meier estimated on-treatment rates at 24 months of 26%, 23%, and 25%, respectively. These findings are aligned with those of others who reported no differences in the effectiveness of first-line ICI therapy according to *KRAS* mutation status [9,10], although a concomitant STK11 mutation was found to be associated with PD-1 inhibitor resistance and worse clinical outcomes [38]. We did not have information regarding *KRAS* mutation subtypes; however, in one large chart review study, responses and duration of benefit with available therapies were similar with *KRAS* G12C mutations versus *KRAS* non-G12C mutations [39]. Moreover, in KN042, patients who had *KRAS*-mutated tumors, including those with the *KRAS* G12C mutation, experienced improved outcomes with pembrolizumab compared with chemotherapy [10]. We note that the first inhibitor of the *KRAS* G12C mutation, sotorasib, was approved in the US in May 2021 for previously treated advanced NSCLC.

The strengths of the current study include the large patient population drawn from a well-curated, well-regarded database, including patients with an ECOG PS of 2, who are usually not eligible for clinical trials. Patient characteristics were well-defined, with known negative status for *EGFR* and *ALK* genomic alterations of nonsquamous tumors and no missing data for tumor PD-L1 expression or patients’ performance status. The median study follow-up was lengthy—almost 3 years.

We acknowledge the possibility of selection bias, for example, because ECOG PS was not consistently documented, so many otherwise eligible patients (26%) were excluded. Moreover, the findings may not be generalizable outside of the Flatiron Health network or for academic centers, as most patients were treated in the community oncology setting. Information was missing for clinically important variables, such as whether brain metastases were pretreated, although observational studies suggest that the survival benefit from pembrolizumab-based therapy may not be inferior for patients with brain metastases [40]. Finally, the *KRAS* subanalysis was limited to the 330 patients with available data, and no data were available regarding *KRAS* G12C mutations or the status of other mutations that have been associated with prognosis, such as *STK11*, *KEAP1*, and *PTEN* gene mutations [41,42].

Further study of well-characterized, real-world patient populations is needed to continue defining the optimal duration of pembrolizumab therapy for advanced NSCLC and the association of rwToT with disease progression and survival. In addition, subgroup analyses of future studies that incorporate key clinical characteristics potentially affecting outcomes with first-line ICI monotherapy could provide useful insights for practicing clinicians. Pembrolizumab-containing regimens remain the standard of care in the first-line treatment of patients with *KRAS*-mutated NSCLC, notwithstanding the recent availability of *KRAS*-targeted therapy.

## 5. Conclusions

Patients with good performance status (ECOG PS 0–1) treated in the US real-world clinical setting for advanced NSCLC, with PD-L1 TPS ≥ 50%, and without *EGFR/ALK/ROS1* genomic alterations, experienced a median rwToT of 7.4 months, similar to the treatment durations observed in clinical trials. Furthermore, 22% remained on first-line pembrolizumab monotherapy at 24 months. For patients with an ECOG PS of 2, the median rwToT was 2.1 months; and 10% remained on first-line pembrolizumab monotherapy at 24 months. We observed no clinically relevant differences in rwToT variables based on *KRAS* mutation status for patients with nonsquamous NSCLC and good performance status. Our findings suggest a long-term benefit of pembrolizumab monotherapy for advanced NSCLC with PD-L1 TPS ≥ 50% in real-world settings in the US, particularly for patients with good performance status at the start of therapy, irrespective of *KRAS* mutation status.

## Figures and Tables

**Table 1:** Baseline characteristics of patients with advanced NSCLC (stage IIIB/C, IV), according to ECOG performance status.

Characteristic	ECOG PS 0–1 *N* = 807	ECOG PS 2 *N* = 237
Men	410 (50.8)	118 (49.8)
Age, median (range), years	72 (38–84)	75 (48–84)
<75 years	484 (60.0)	116 (48.9)
≥75 years	323 (40.0)	121 (51.1)
Race data available, N	724	209
White ^1^	566 (78.2)	159 (76.1)
Black ^1^	68 (9.4)	23 (11.0)
Asian ^1^	20 (2.8)	1 (0.5)
Other race ^1^	70 (9.7)	26 (12.4)
Current/former smoker	747 (92.6)	226 (95.4)
No smoking history	60 (7.4)	11 (4.6)
Charlson comorbidity index score, mean (SD)	4.9 (3.1)	5.4 (3.1)
Median (range)	3 (0–14)	4 (2–12)
NSCLC histology		
Nonsquamous	544 (67.4)	170 (71.7)
Squamous	220 (27.3)	57 (24.1)
NSCLC histology NOS	43 (5.3)	10 (4.2)
NSCLC first diagnosed at stage IV	566 (70.1)	178 (75.1)
*KRAS* mutation status (nonsquamous only), N	544	170
Positive ^2^	164 (30.1)	49 (28.8)
Wild-type	166 (30.5)	48 (28.2)
Indeterminate, unknown, pending, untested	214 (39.3)	73 (42.9)
Record of brain metastases ^3^	81 (10.0)	27 (11.4)

Data are *n* (%) unless otherwise noted. Percentages may not add up to 100 because of rounding. ^1^ Percentages for race represent the percentages of patients with available data. ^2^ Positive biomarker results at any time (“ever positive”) were included. ^3^ Information about prior treatment of brain metastases was not available. ECOG PS: Eastern Cooperative Oncology Group performance status; NSCLC histology NOS: non-small cell lung cancer histology not otherwise specified.

**Table 2:** Real-world time on treatment with first-line pembrolizumab monotherapy for patients with advanced NSCLC, according to ECOG performance status.

Variable	ECOG PS 0–1 *N* = 807	ECOG PS 2 *N* = 237
Theoretical follow-up, median (range), mo ^1^	34.1 (12.0–52.7)	33.5 (12.2–52.7)
Patient follow-up, median (range), mo ^1^	17.4 (<0.1–52.6)	5.7 (<0.1–51.5)
Discontinued pembrolizumab, *n* (%)	628 (77.8)	207 (87.3)
rwToT, median (95% CI), mo	7.4 (6.3–8.1)	2.1 (1.4–2.8)
Restricted mean rwToT (95% CI), mo		
Restricted to 12 months	7.0 (6.7–7.3)	4.3 (3.7–5.0)
Restricted to 24 months	10.3 (9.7–11.0)	5.9 (4.9–7.0) (Lognormal)
Restricted to 36 months	12.4 (11.5–13.3) (Weibull)	7.4 (6.0–9.0) (Lognormal)
Restricted to 48 months	13.6 (12.6–14.8) (Weibull)	8.5 (6.9–10.6) (Lognormal)
On-treatment rate, % (95% CI) ^2^		
At 12 months	36.0 (32.6–39.3)	20.8 (15.7–26.3)
At 24 months	22.1 (19.1–25.3)	9.9 (6.1–14.6)
At 36 months	13.7 (10.9–16.9)	6.1 (2.8–11.1)
At 48 months	9.8 (6.7–13.6)	n/a

^1^ Theoretical follow-up was defined as the duration of follow-up from first-line therapy initiation to database cutoff (31 March 2021). Patient follow-up was defined as time from first-line therapy initiation to the date of death or data cutoff, whichever occurred first. ^2^ On-treatment rates were based on Kaplan–Meier estimates. mo: months; n/a: not assessable; rwToT: real-world time on treatment.

**Table 3:** Real-world time on treatment with first-line pembrolizumab monotherapy for patients with ECOG performance status 0–1, according to *KRAS* mutation status.

Variable	All Nonsquamous *N* = 544	*KRAS* Positive *N* = 164	*KRAS* Wild-Type *N* = 166	*KRAS* Unknown *N* = 214
Pembrolizumab rwToT				
Discontinued pembrolizumab, *n* (%)	410 (75.4)	126 (76.8)	131 (78.9)	153 (71.5)
rwToT, median (95% CI), mo	7.6 (6.3–8.8)	7.6 (6.3–10.6)	7.0 (5.3–9.3)	7.9 (4.8–10.8)
Restricted mean rwToT (95% CI), mo				
Restricted to 12 months	7.1 (6.7–7.5)	7.4 (6.7–8.1)	6.9 (6.3–7.7)	7.0 (6.3–7.7)
Restricted to 24 months	10.7 (9.9–11.5)	11.3 (9.9–12.8)	10.3 (9.0–11.8)	10.5 (9.3–11.8)
Restricted to 36 months	13.0 (11.9–14.1) (Weibull)	13.5 (11.6–15.6) (Weibull)	12.2 (10.4–14.3) (Weibull)	13.2 (11.4–15.2) (Weibull)
Restricted to 48 months	14.5 (13.1–16.0) (Weibull)	14.9 (12.6–17.7) (Weibull)	13.4 (11.2–16.0) (Weibull)	15.0 (12.7–17.6) (Weibull)
On-treatment rate, % (95% CI) ^1^				
At 12 months	37.1 (33.0–41.3)	38.3 (30.8–45.8)	35.1 (27.8–42.5)	37.9 (31.2–44.7)
At 24 months	24.6 (20.7–28.6)	25.7 (18.8–33.1)	23.0 (16.4–30.3)	25.1 (18.9–31.7)
At 36 months	16.5 (12.8–20.6)	17.7 (11.2–25.4)	12.5 (6.9–19.8)	19.1 (13.2–26.0)
At 48 months	10.6 (6.5–15.8)	n/a	9.4 (4.4–16.6)	17.5 (11.5–24.6)

^1^ On-treatment rates were based on Kaplan–Meier estimates. mo: months; n/a: not assessable; rwToT: real-world time on treatment.

**Table 4:** Subsequent systemic anticancer therapy regimens.

Regimen by Treatment Line ^1^	ECOG 0–1 *N* = 807	ECOG 2 *N* = 237
Systemic Therapy Line 2	263 (32.6)	39 (16.5)
Anti-PD-1/PD-L1-based therapies	105 (39.9)	11 (28.2)
Anti-PD-1/PD-L1 monotherapy	36 (34.3)	3 (27.3)
Anti-PD-1/PD-L1 combination therapy	69 (65.7)	8 (72.7)
Anti-VEGF-based therapies	25 (9.5)	4 (10.3)
Platinum-based chemotherapy combinations	88 (33.5)	17 (43.6)
Nonplatinum-based chemotherapy combinations	3 (1.1)	0
Single-agent chemotherapy	29 (11.0)	6 (15.4)
Other therapy	13 (4.9)	1 (2.6)
Systemic Therapy Line 3	91 (11.3)	10 (4.2)
Anti-PD-1/PD-L1-based therapies	34 (37.4)	4 (40.0)
Anti-PD-1/PD-L1 monotherapy	15 (44.1)	2 (50.0)
Anti-PD-1/PD-L1 combination therapy	19 (55.9)	2 (50.0)
Anti-VEGF-based therapies	12 (13.2)	2 (20.0)
Platinum-based chemotherapy combinations	13 (14.3)	0
Nonplatinum-based chemotherapy combinations	1 (1.1)	0
Single-agent chemotherapy	24 (26.4)	3 (30.0)
Other therapy	7 (7.7)	1 (10.0)
Systemic Therapy Line 4	24 (3.0)	4 (1.7)
Anti-PD-1/PD-L1-based therapies	5 (20.8)	1 (25.0)
Anti-PD-1/PD-L1 monotherapy	1 (20.0)	0
Anti-PD-1/PD-L1 combination therapy	4 (80.0)	1 (100)
Anti-VEGF-based therapies	4 (16.7)	0
Platinum-based chemotherapy combinations	5 (20.8)	0
Single-agent chemotherapy	8 (33.3)	1 (25.0)
Other therapy	2 (8.3)	2 (50.0)
Systemic Therapy Line 5	7 (0.9) ^2^	1 (0.4) ^3^
Anti-PD-1/PD-L1-based therapies	3 (42.9)	0
Anti-PD-1/PD-L1 monotherapy	2 (66.7)	0
Anti-PD-1/PD-L1 combination therapy	1 (33.3)	0
Anti-VEGF-based therapies	1 (14.3)	0
Platinum-based chemotherapy combinations	1 (14.3)	0
Single-agent chemotherapy	1 (14.3)	1 (100)
Other therapy	1 (14.3)	0

^1^ Drug regimen classes are shown as a percentage of the relevant treatment line, with anti-PD-1/PD-L1 monotherapy and combination therapy shown as a percentage of the anti-PD-1/PD-L1-based therapy regimen class. Mutually exclusive regimen classes were assigned in hierarchical order as shown, beginning with anti-PD-1/PD-L1-based therapy. Data are *n* (%), and percentages may not total 100 because of rounding. ^2^ Three patients continued to line 6 and received anti-PD-1/PD-L1 combination therapy, anti-VEGF-based therapy, and other therapy; and one patient received other therapy in line 7. ^3^ One patient continued to line 6 and received single-agent chemotherapy. PD-1/PD-L1: programmed death 1/PD-ligand 1; VEGF: vascular endothelial growth factor.

## Data Availability

The data that support the findings of this study have been originated by Flatiron Health, Inc. These deidentified data may be made available upon request and are subject to a license agreement with Flatiron Health; interested researchers should contact DataAccess@flatiron.com to determine licensing terms.

## Supplementary Materials

The following are available online at https://www.mdpi.com/article/10.3390/cancers14041041/s1, Table S1. Additional characteristics of patients with advanced NSCLC (stage IIIB/C, IV), according to ECOG performance status; Table S2. Distribution of the number of administered cycles of first-line pembrolizumab for patients with advanced NSCLC, according to ECOG performance status, among patients with at least 2 years of theoretical follow-up; Table S3. Baseline characteristics of patients with initial NSCLC diagnosis made at stage IV, according to ECOG performance status; Table S4. Real-world time on treatment with pembrolizumab for patients with initial NSCLC diagnosis made at stage IV, according to ECOG performance status; Table S5. Subsequent systemic anticancer therapy regimens; Figure S1. Kaplan–Meier plot depicting real-world time on treatment (rwToT) with pembrolizumab for patients with initial NSCLC diagnosis made at stage IV, according to ECOG performance status.

## References

[1] National Cancer Institute: Surveillance, E. and End Results Program (SEER) Cancer Stat Facts: Lung and Bronchus Cancer. https://seer.cancer.gov/statfacts/html/lungb.html.

[2] Howlader N., Forjaz G., Mooradian M.J., Meza R., Kong C.Y., Cronin K.A., Mariotto A.B., Lowy D.R., Feuer E.J. (2020). The effect of advances in lung-cancer treatment on population mortality. N. Engl. J. Med..

[3] Siegel R.L., Miller K.D., Fuchs H.E., Jemal A. (2021). Cancer Statistics, 2021. CA Cancer J. Clin..

[4] Grant M.J., Herbst R.S., Goldberg S.B. (2021). Selecting the optimal immunotherapy regimen in driver-negative metastatic NSCLC. Nat. Rev. Clin. Oncol..

[5] Steuer C.E., Ramalingam S.S. (2021). Advances in immunotherapy and implications for current practice in non-small-cell lung cancer. JCO Oncol. Pract..

[6] Calles A., Riess J.W., Brahmer J.R. (2020). Checkpoint blockade in lung cancer with driver mutation: Choose the road wisely. Am. Soc. Clin. Oncol. Educ. Book.

[7] National Comprehensive Cancer Network (NCCN) NCCN Clinical Practice Guidelines in Oncology: Non-Small Cell Lung Cancer, Version 1.2022—7 December 2021. https://www.nccn.org/professionals/physician_gls/pdf/nscl.pdf.

[8] El Osta B., Behera M., Kim S., Berry L.D., Sica G., Pillai R.N., Owonikoko T.K., Kris M.G., Johnson B.E., Kwiatkowski D.J. (2019). Characteristics and outcomes of patients with metastatic KRAS-mutant lung adenocarcinomas: The Lung Cancer Mutation Consortium experience. J. Thorac. Oncol..

[9] Mazieres J., Drilon A., Lusque A., Mhanna L., Cortot A.B., Mezquita L., Thai A.A., Mascaux C., Couraud S., Veillon R. (2019). Immune checkpoint inhibitors for patients with advanced lung cancer and oncogenic driver alterations: Results from the IMMUNOTARGET registry. Ann. Oncol..

[10] Herbst R.S., Lopes G., Kowalski D.M., Kasahara K., Wu Y.L., De Castro G., Cho B.C., Turna H.Z., Cristescu R., Aurora-Garg D. (2019). LBA4 Association of KRAS mutational status with response to pembrolizumab monotherapy given as first-line therapy for PD-L1-positive advanced non-squamous NSCLC in Keynote-042. Ann. Oncol..

[11] Noordhof A.L., Damhuis R.A.M., Hendriks L.E.L., de Langen A.J., Timens W., Venmans B.J.W., van Geffen W.H. (2021). Prognostic impact of KRAS mutation status for patients with stage IV adenocarcinoma of the lung treated with first-line pembrolizumab monotherapy. Lung Cancer.

[12] Reck M., Rodriguez-Abreu D., Robinson A.G., Hui R., Csoszi T., Fulop A., Gottfried M., Peled N., Tafreshi A., Cuffe S. (2016). Pembrolizumab versus chemotherapy for PD-L1-positive non-small-cell lung cancer. N. Engl. J. Med..

[13] Reck M., Rodriguez-Abreu D., Robinson A.G., Hui R., Csoszi T., Fulop A., Gottfried M., Peled N., Tafreshi A., Cuffe S. (2019). Updated analysis of KEYNOTE-024: Pembrolizumab versus platinum-based chemotherapy for advanced non-small-cell lung cancer with PD-L1 tumor proportion score of 50% or greater. J. Clin. Oncol..

[14] Reck M., Rodriguez-Abreu D., Robinson A.G., Hui R., Csoszi T., Fulop A., Gottfried M., Peled N., Tafreshi A., Cuffe S. (2021). Five-year outcomes with pembrolizumab versus chemotherapy for metastatic non-small-cell lung cancer with PD-L1 tumor proportion score ≥ 50%. J. Clin. Oncol..

[15] Miller R.S., Wong J.L. (2018). Using oncology real-world evidence for quality improvement and discovery: The case for ASCO’s CancerLinQ. Future Oncol..

[16] Berger M.L., Curtis M.D., Smith G., Harnett J., Abernethy A.P. (2016). Opportunities and challenges in leveraging electronic health record data in oncology. Future Oncol..

[17] Friends of Cancer Research Establishing a Framework to Evaluate Real-World Endpoints. https://www.focr.org/publications/establishing-framework-evaluate-real-world-endpoints.

[18] Blumenthal G.M., Gong Y., Kehl K., Mishra-Kalyani P., Goldberg K.B., Khozin S., Kluetz P.G., Oxnard G.R., Pazdur R. (2019). Analysis of time-to-treatment discontinuation of targeted therapy, immunotherapy, and chemotherapy in clinical trials of patients with non-small-cell lung cancer. Ann. Oncol..

[19] Stewart M., Norden A.D., Dreyer N., Henk H.J., Abernethy A.P., Chrischilles E., Kushi L., Mansfield A.S., Khozin S., Sharon E. (2019). An exploratory analysis of real-world end points for assessing outcomes among immunotherapy-treated patients with advanced non-small-cell lung cancer. JCO Clin. Cancer Inform..

[20] Khozin S., Miksad R.A., Adami J., Boyd M., Brown N.R., Gossai A., Kaganman I., Kuk D., Rockland J.M., Pazdur R. (2019). Real-world progression, treatment, and survival outcomes during rapid adoption of immunotherapy for advanced non-small cell lung cancer. Cancer.

[21] Kehl K.L., Riely G.J., Lepisto E.M., Lavery J.A., Warner J.L., LeNoue-Newton M.L., Sweeney S.M., Rudolph J.E., Brown S., Yu C. (2021). Correlation between surrogate end points and overall survival in a multi-institutional clinicogenomic cohort of patients with non-small cell lung or colorectal cancer. JAMA Netw. Open.

[22] Velcheti V., Chandwani S., Chen X., Pietanza M.C., Burke T. (2019). First-line pembrolizumab monotherapy for metastatic PD-L1-positive NSCLC: Real-world analysis of time on treatment. Immunotherapy.

[23] Flatiron Health Flatiron Health Database. https://flatiron.com/real-world-evidence/.

[24] Abernethy A.P., Gippetti J., Parulkar R., Revol C. (2017). Use of electronic health record data for quality reporting. J. Oncol. Pract..

[25] Griffith S.D., Tucker M., Bowser B., Calkins G., Chang C.J., Guardino E., Khozin S., Kraut J., You P., Schrag D. (2019). Generating real-world tumor burden endpoints from electronic health record data: Comparison of RECIST, radiology-anchored, and clinician-anchored approaches for abstracting real-world progression in non-small cell lung cancer. Adv. Ther..

[26] Khan N.F., Perera R., Harper S., Rose P.W. (2010). Adaptation and validation of the Charlson Index for Read/OXMIS coded databases. BMC Fam. Pract..

[27] Velcheti V., Chandwani S., Chen X., Pietanza M.C., Piperdi B., Burke T. (2019). Outcomes of first-line pembrolizumab monotherapy for PD-L1-positive (TPS ≥ 50%) metastatic NSCLC at US oncology practices. Immunotherapy.

[28] Pocock S.J., Clayton T.C., Altman D.G. (2002). Survival plots of time-to-event outcomes in clinical trials: Good practice and pitfalls. Lancet.

[29] Latimer N.R. (2013). Survival analysis for economic evaluations alongside clinical trials--extrapolation with patient-level data: Inconsistencies, limitations, and a practical guide. Med. Decis. Mak..

[30] Mok T.S.K., Wu Y.L., Kudaba I., Kowalski D.M., Cho B.C., Turna H.Z., Castro G., Srimuninnimit V., Laktionov K.K., Bondarenko I. (2019). Pembrolizumab versus chemotherapy for previously untreated, PD-L1-expressing, locally advanced or metastatic non-small-cell lung cancer (KEYNOTE-042): A randomised, open-label, controlled, phase 3 trial. Lancet.

[31] Khozin S., Abernethy A.P., Nussbaum N.C., Zhi J., Curtis M.D., Tucker M., Lee S.E., Light D.E., Gossai A., Sorg R.A. (2018). Characteristics of real-world metastatic non-small cell lung cancer patients treated with nivolumab and pembrolizumab during the year following approval. Oncologist.

[32] Jenei K., Meyers D.E., Prasad V. (2021). The inclusion of women in global oncology drug trials over the past 20 years. JAMA Oncol..

[33] Murthy V.H., Krumholz H.M., Gross C.P. (2004). Participation in cancer clinical trials: Race-, sex-, and age-based disparities. JAMA.

[34] Stenehjem D., Lubinga S., Betts K.A., Tang W., Jenkins M., Yuan Y., Hartman J., Rao S., Lam J., Waterhouse D. (2021). Treatment patterns in patients with metastatic non-small-cell lung cancer in the era of immunotherapy. Future Oncol..

[35] Waterhouse D., Lam J., Betts K.A., Yin L., Gao S., Yuan Y., Hartman J., Rao S., Lubinga S., Stenehjem D. (2021). Real-world outcomes of immunotherapy-based regimens in first-line advanced non-small cell lung cancer. Lung Cancer.

[36] Herbst R.S., Giaccone G., de Marinis F., Reinmuth N., Vergnenegre A., Barrios C.H., Morise M., Felip E., Andric Z., Geater S. (2020). Atezolizumab for first-line treatment of PD-L1-selected patients with NSCLC. N. Engl. J. Med..

[37] Sezer A., Kilickap S., Gumus M., Bondarenko I., Ozguroglu M., Gogishvili M., Turk H.M., Cicin I., Bentsion D., Gladkov O. (2021). Cemiplimab monotherapy for first-line treatment of advanced non-small-cell lung cancer with PD-L1 of at least 50%: A multicentre, open-label, global, phase 3, randomised, controlled trial. Lancet..

[38] Skoulidis F., Goldberg M.E., Greenawalt D.M., Hellmann M.D., Awad M.M., Gainor J.F., Schrock A.B., Hartmaier R.J., Trabucco S.E., Gay L. (2018). STK11/LKB1 Mutations and PD-1 inhibitor resistance in KRAS-mutant lung adenocarcinoma. Cancer Discov..

[39] Arbour K.C., Rizvi H., Plodkowski A.J., Hellmann M.D., Knezevic A., Heller G., Yu H.A., Ladanyi M., Kris M.G., Arcila M.E. (2021). Treatment outcomes and clinical characteristics of patients with KRAS-G12C-mutant non-small cell lung cancer. Clin. Cancer Res..

[40] Sun L., Davis C.W., Hwang W.T., Jeffries S., Sulyok L.F., Marmarelis M.E., Singh A.P., Berman A.T., Feigenberg S.J., Levin W. (2021). Outcomes in patients with non-small-cell lung cancer with brain metastases treated with pembrolizumab-based therapy. Clin. Lung Cancer.

[41] Di Federico A., De Giglio A., Parisi C., Gelsomino F. (2021). STK11/LKB1 and KEAP1 mutations in non-small cell lung cancer: Prognostic rather than predictive?. Eur. J. Cancer.

[42] Gkountakos A., Sartori G., Falcone I., Piro G., Ciuffreda L., Carbone C., Tortora G., Scarpa A., Bria E., Milella M. (2019). PTEN in lung cancer: Dealing with the problem, building on new knowledge and turning the game around. Cancers.

