# Supplementary material for: Real-World Time on Treatment with First-Line Pembrolizumab Monotherapy for Advanced NSCLC with PD-L1 Expression ≥ 50%: 3-Year Follow-Up Data
Source: Cancers (Basel). 2022 Feb 18;14(4):1041. doi: 10.3390/cancers14041041 (PMC8870405; doi:10.3390/cancers14041041)
Supplement: Supplementary file 1 [file cancers-14-01041-s001.zip › cancers-1568901-supplementary.pdf]

cancers MDPI

Table S1. Additional characteristics of patients with advanced NSCLC (stage IIIB/C, IV), according to ECOG performance status.

Characteristic	ECOG PS 0–1 *N* = 807	ECOG PS 2 *N* = 237
US CB region, data available, *N*	*780*	*227*
Northeast [1]	145 (18.6)	45 (19.8)
Midwest [1]	174 (22.3)	43 (18.9)
South [1]	353 (45.3)	119 (52.4)
West [1]	108 (13.8)	20 (8.8)
Community oncology clinic	789 (97.8)	230 (97.0)
Academic oncology clinic	18 (2.2)	7 (3.0)
Index year		
2016	18 (2.2)	11 (4.6)
2017	275 (34.1)	69 (29.1)
2018	241 (29.9)	75 (31.6)
2019	209 (25.9)	67 (28.3)
2020	64 (7.9)	15 (6.3)
BRAF mutation status (nonsquamous only), *N*	*544*	*170*
Positive [2]	37 (6.8)	8 (4.7)
Wild type	338 (62.1)	102 (60.0)
Indeterminate, unknown, pending, untested	169 (31.1)	60 (35.3)

Data are *n* (%) unless otherwise noted. Percentages may not add up to 100% because of rounding.
[1] Percentages for US CB region represent the percentages of patients with available data.
[2] Positive biomarker results at any time ("ever positive") were included.
US CB: United States Census Bureau.

Table S2. Distribution of the number of administered cycles of first-line pembrolizumab for patients with advanced NSCLC, according to ECOG performance status, among patients with at least 2 years of theoretical follow-up [1]

**Variable**	**ECOG PS 0–1**<br>***N* = 587**	**ECOG PS 2**<br>***N* = 174**
Number of cycles, mean (SD)	16.0 (16.3)	9.5 (13.4)
Median (range)	10.0 (1–73)	3.5 (1–68)
Number of cycles, *n* (%) [2]		
1	55 (9.4)	50 (28.7)
2	39 (6.6)	18 (10.3)
3	41 (7.0)	19 (10.9)
4	41 (7.0)	10 (5.7)
5	33 (5.6)	11 (6.3)
6–17	189 (32.2)	34 (19.5)
18–34	97 (16.5)	18 (10.3)
≥35	92 (15.7)	14 (8.0)

[1] Patients initiating first-line pembrolizumab on or before 31 March 2019 (data cutoff, 31 March 2021).

[2] 35 (6.0%) and 3 (1.7%) in PS 0–1 and PS 2 cohorts, respectively, received the pembrolizumab 400 mg dosage.

Table S3. Baseline characteristics of patients with initial NSCLC diagnosis made at stage IV, according to ECOG performance status.

Characteristic	ECOG PS 0–1<br>*N* = 566	ECOG PS 2<br>*N* = 178
Men	298 (52.7)	89 (50.0)
Age, median (range), yr	71 (38–84)	75 (48–84)
<75 yr	359 (63.4)	88 (49.4)
≥75 yr	207 (36.6)	90 (50.6)
Race data available, *N*	*505*	*153*
White [1]	383 (75.8)	108 (70.6)
Black [1]	49 (9.7)	21 (13.7)
Asian [1]	17 (3.4)	1 (0.7)
Other race [1]	56 (11.1)	23 (15.0)
Current/former smoker	524 (92.6)	169 (94.9)
No smoking history	42 (7.4)	9 (5.1)
CCI score, mean (SD)	5.1 (3.1)	5.5 (3.2)
Median (range)	4 (0-13)	6 (2-11)
NSCLC histology		
Nonsquamous	405 (71.6)	132 (74.2)
Squamous	133 (23.5)	39 (21.9)
NSCLC histology NOS	28 (4.9)	7 (3.9)
KRAS mutation status (nonsquamous only), *N*	*405*	*132*
Positive [2]	107 (26.4)	40 (30.3)
Wild type	125 (30.9)	34 (25.8)
Indeterminate, unknown, pending, untested	173 (42.7)	58 (43.9)
Record of brain metastases [3]	69 (12.2)	23 (12.9)

Data are *n* (%) unless otherwise noted. Percentages may not add up to 100 because of rounding.
[1] Percentages for race represent the percentages of patients with available data.
[2] Positive biomarker results at any time ("ever positive") were included.
[3] Information about prior treatment of brain metastases was not available.
CCI: Charlson comorbidity index; ECOG: Eastern Cooperative Oncology Group; NSCLC histology NOS: non-small cell lung cancer histology not otherwise specified.

Table S4. Real-world time on treatment with first-line pembrolizumab monotherapy for patients with initial NSCLC diagnosis made at stage IV, according to ECOG performance status.

Variable	ECOG PS 0–1<br>*N* = 566	ECOG PS 2<br>*N* = 178
Theoretical follow-up, median (range), mo [1]	35.1 (12.0–52.7)	33.3 (12.2–52.3)
Patient follow-up, median (range), mo [1]	16.5 (<0.1–52.6)	4.3 (<0.1–51.5)
Discontinued pembrolizumab, *n* (%)	441 (77.9)	157 (88.2)
rwToT, median (95% CI), mo	6.5 (5.6–7.6)	1.6 (0.7–2.8)
Restricted mean rwToT (95% CI), mo		
Restricted to 12 months	6.8 (6.4–7.2)	3.9 (3.3–4.7)
Restricted to 24 months	10.0 (9.2–10.7)	5.2 (4.2–6.5)<br>[Lognormal]
Restricted to 36 months	12.1 (11.1–13.2)<br>[Weibull]	6.5 (5.1–8.3)<br>[Lognormal]
Restricted to 48 months	13.3 (12.1–14.8)<br>[Weibull]	7.5 (5.7–9.8)<br>[Lognormal]
On-treatment rate, % (95% CI) [2]		
At 12 months	34.5 (30.5–38.5)	19.4 (13.8–25.7)
At 24 months	21.2 (17.7–25.0)	7.7 (4.0–13.0)
At 36 months	14.3 (11.0–18.1)	6.6 (3.1–11.9)
At 48 months	10.4 (6.7–15.1)	n/a

[1] Theoretical follow-up was defined as the duration of follow-up from first-line therapy initiation to database cutoff (31 March 2021). Patient follow-up was defined as time from first-line therapy initiation to the date of death or data cutoff, whichever occurred first.
[2] On-treatment rates were based on Kaplan-Meier estimates.
mo: months; n/a: not assessable; rwToT: real-world time on treatment.

Table S5. Subsequent systemic anticancer therapy regimens

Regimen by treatment line [1]	ECOG 0–1 *N* = 807	ECOG 2 *N* = 237
**Systemic Therapy Line 2**	**263 (32.6)**	**39 (16.5)**
Line 2: Anti-PD-1/PD-L1-based therapies	*105 (39.9)*	*11 (28.2)*
Carboplatin, pembrolizumab, pemetrexed	27 (25.7)	2 (18.2)
Pembrolizumab	25 (23.8)	3 (27.3)
Carboplatin, paclitaxel, pembrolizumab	12 (11.4)	2 (18.2)
Nivolumab	8 (7.6)	0
Pembrolizumab, pemetrexed	5 (4.8)	1 (9.1)
Carboplatin, paclitaxel protein-bound, pembrolizumab	2 (1.9)	1 (9.1)
Atezolizumab	3 (2.9)	0
Anastrozole, pembrolizumab	2 (1.9)	0
Bevacizumab-awwb, pembrolizumab	1 (1.0)	0
Exemestane, pembrolizumab	2 (1.9)	0
Imatinib, pembrolizumab	1 (1.0)	0
Paclitaxel, pembrolizumab	1 (1.0)	1 (9.1)
Anastrozole, carboplatin, pembrolizumab, pemetrexed	1 (1.0)	0
Atezolizumab, carboplatin, paclitaxel	1 (1.0)	0
Atezolizumab, carboplatin, paclitaxel protein-bound	1 (1.0)	0
Bevacizumab, pembrolizumab	1 (1.0)	0
Bevacizumab, pembrolizumab, pemetrexed	1 (1.0)	0
Cabozantinib, crizotinib, pembrolizumab	1 (1.0)	0
Capecitabine, pembrolizumab	1 (1.0)	0
Carboplatin, docetaxel, pembrolizumab	1 (1.0)	0
Carboplatin, gemcitabine, pembrolizumab, pemetrexed	1 (1.0)	0
Carboplatin, ipilimumab, nivolumab, pemetrexed	1 (1.0)	0
Carboplatin, pembrolizumab	1 (1.0)	0
Hydroxyurea, pembrolizumab	1 (1.0)	0
Ipilimumab, nivolumab	0	1 (9.1)
Ipilimumab, nivolumab, pembrolizumab	1 (1.0)	0
Letrozole, pembrolizumab	1 (1.0)	0
Medroxyprogesterone, pembrolizumab	1 (1.0)	0
Niraparib, pembrolizumab	1 (1.0)	0
Line 2: Anti-VEGF-based therapies	*25 (9.5)*	*4 (10.3)*
Bevacizumab, carboplatin, pemetrexed	16 (64.0)	2 (50.0)
Docetaxel, ramucirumab	2 (8.0)	1 (25.0)
Bevacizumab-awwb, carboplatin, pemetrexed	3 (12.0)	1 (25.0)
Bevacizumab, cisplatin, pemetrexed	1 (4.0)	0
Bevacizumab, gemcitabine	1 (4.0)	0
Bevacizumab-bvzr, carboplatin, paclitaxel	1 (4.0)	0
Bevacizumab-bvzr, fluorouracil, leucovorin, oxaliplatin	1 (4.0)	0
Line 2: Platinum-based chemotherapy combinations	*88 (33.5)*	*17 (43.6)*
Carboplatin, pemetrexed	41 (46.6)	6 (35.3)
Carboplatin, paclitaxel	21 (23.9)	3 (17.6)
Carboplatin, paclitaxel protein-bound	14 (15.9)	5 (29.4)
Carboplatin, gemcitabine	9 (10.2)	2 (11.8)

Carboplatin, docetaxel	0	1 (5.9)
Carboplatin, etoposide	1 (1.1)	0
Carboplatin, etoposide, paclitaxel	1 (1.1)	0
Cisplatin, gemcitabine	1 (1.1)	0
Line 2: Non-platinum-based chemo combinations	*3 (1.1)*	*0*
Docetaxel, gemcitabine	1 (33.3)	0
Abiraterone, pemetrexed	1 (33.3)	0
Gemcitabine, paclitaxel	1 (33.3)	0
Line 2: Single agent chemotherapy	*29 (11.0)*	*6 (15.4)*
Pemetrexed	14 (48.3)	2 (33.3)
Docetaxel	8 (27.6)	3 (50.0)
Gemcitabine	4 (13.8)	1 (16.7)
Vinorelbine	2 (6.9)	0
Paclitaxel	1 (3.4)	0
Line 2: Other therapy	*13 (4.9)*	*1 (2.6)*
Dabrafenib, trametinib	4 (30.8)	0
Crizotinib	1 (7.7)	0
Capecitabine	1 (7.7)	0
Capmatinib	2 (15.4)	0
Ado-trastuzumab emtansine	1 (7.7)	0
Alectinib	1 (7.7)	0
Cyclophosphamide, rituximab, vincristine	1 (7.7)	0
Leuprolide	1 (7.7)	0
Methotrexate	1 (7.7)	0
Olaparib	0	1 (100)
**Systemic Therapy Line 3**	**91 (11.3)**	**10 (4.2)**
Line 3: Anti-PD-1/PD-L1-based therapies	*34 (37.4)*	*4 (40.0)*
Pembrolizumab	7 (20.6)	1 (25.0)
Nivolumab	5 (14.7)	0
Atezolizumab	3 (8.8)	1 (25.0)
Carboplatin, pembrolizumab, pemetrexed	3 (8.8)	0
Carboplatin, paclitaxel, pembrolizumab	3 (8.8)	0
Docetaxel, pembrolizumab	1 (2.9)	1 (25.0)
Pembrolizumab, pemetrexed	2 (5.9)	0
Anastrozole, capecitabine, pembrolizumab	1 (2.9)	0
Atezolizumab, bevacizumab-awwb, carboplatin, paclitaxel	1 (2.9)	0
Bevacizumab-awwb, carboplatin, pembrolizumab, pemetrexed	1 (2.9)	0
Capecitabine, pembrolizumab	1 (2.9)	0
Carboplatin, gemcitabine, pembrolizumab	1 (2.9)	0
Docetaxel, gemcitabine, nivolumab	1 (2.9)	0
Fluorouracil, irinotecan, pembrolizumab	1 (2.9)	0
Ipilimumab, nivolumab	0	1 (25.0)
Nivolumab, temozolomide	1 (2.9)	0
Paclitaxel, pembrolizumab	1 (2.9)	0
Pembrolizumab, rucaparib	1 (2.9)	0
Line 3: Anti-VEGF-based therapies	*12 (13.2)*	*2 (20.0)*
Docetaxel, ramucirumab	6 (50.0)	1 (50.0)
Bevacizumab-awwb, carboplatin, pemetrexed	1 (8.3)	0
Bevacizumab-awwb, pemetrexed	1 (8.3)	0

Bevacizumab	1 (8.3)	0
Bevacizumab, carboplatin, gemcitabine	1 (8.3)	0
Bevacizumab, pemetrexed	0	1 (50.0)
Bevacizumab-bvzr, carboplatin, pemetrexed	1 (8.3)	0
Paclitaxel, ramucirumab	1 (8.3)	0
Line 3: Platinum-based chemotherapy combinations	*13 (14.3)*	*0*
Carboplatin, paclitaxel	6 (46.2)	0
Carboplatin, pemetrexed	4 (30.8)	0
Carboplatin, docetaxel	1 (7.7)	0
Carboplatin, gemcitabine	1 (7.7)	0
Carboplatin, hydroxyurea, pemetrexed	1 (7.7)	0
Line 3: Non-platinum-based chemo combinations	*1 (1.1)*	*0*
Docetaxel, paclitaxel protein-bound	1 (100)	0
Line 3: Single agent chemotherapy	*24 (26.4)*	*3 (30.0)*
Docetaxel	7 (29.2)	2 (66.7)
Gemcitabine	9 (37.5)	0
Pemetrexed	4 (16.7)	0
Vinorelbine	4 (16.7)	0
Paclitaxel protein-bound	0	1 (33.3)
Line 3: Other therapy	*7 (7.7)*	*1 (10.0)*
Durvalumab	2 (28.6)	1 (100)
Afatinib	1 (14.3)	0
Alectinib, carboplatin, paclitaxel	1 (14.3)	0
Brigatinib	1 (14.3)	0
Erlotinib	1 (14.3)	0
Osimertinib	1 (14.3)	0
**Systemic Therapy Line 4**	**24 (3.0)**	**4 (1.7)**
Line 4: Anti-PD-1/PD-L1-based therapies	*5 (20.8)*	*1 (25.0)*
Gemcitabine, pembrolizumab	1 (20.0)	1 (100)
Atezolizumab	1 (20.0)	0
Docetaxel, irinotecan, pembrolizumab	1 (20.0)	0
Fluorouracil, leucovorin, pembrolizumab	1 (20.0)	0
Ipilimumab, nivolumab	1 (20.0)	0
Line 4: Anti-VEGF-based therapies	*4 (16.7)*	*0*
Docetaxel, ramucirumab	2 (50.0)	0
Bevacizumab-awwb, carboplatin, paclitaxel protein-bound	1 (25.0)	0
Bevacizumab-bvzr	1 (25.0)	0
Line 4: Platinum-based chemotherapy combinations	*5 (20.8)*	*0*
Carboplatin, etoposide	1 (20.0)	0
Carboplatin, gemcitabine	1 (20.0)	0
Carboplatin, gemcitabine, temozolomide	1 (20.0)	0
Carboplatin, paclitaxel	1 (20.0)	0
Cisplatin, gemcitabine	1 (20.0)	0
Line 4: Single agent chemotherapy	*8 (33.3)*	*1 (25.0)*
Gemcitabine	3 (37.5)	1 (100)
Vinorelbine	3 (37.5)	0
Docetaxel	1 (12.5)	0
Pemetrexed	1 (12.5)	0
Line 4: Other therapy	*2 (8.3)*	*2 (50.0)*
Capmatinib	0	1 (50.0)

Dabrafenib, trametinib	0	1 (50.0)
Leuprolide	1 (50.0)	0
Regorafenib	1 (50.0)	0
**Systemic Therapy Line 5**	**7 (0.9)**	**1 (0.4)**
Line 5: Anti-PD-1/PD-L1-based therapies	*3 (42.9)*	*0*
Atezolizumab	1 (33.3)	0
Capecitabine, epirubicin, pembrolizumab	1 (33.3)	0
Nivolumab	1 (33.3)	0
Line 5: Anti-VEGF-based therapies	*1 (14.3)*	*0*
Ramucirumab	1 (100)	0
Line 5: Platinum-based chemotherapy combinations	*1 (14.3)*	*0*
Carboplatin, paclitaxel	1 (100)	0
Line 5: Single agent chemotherapy	*1 (14.3)*	*1 (100)*
Gemcitabine	1 (100)	0
Docetaxel	0	1 (100)
Line 5: Other therapy	*1 (14.3)*	*0*
Erlotinib	1 (100)	0
**Systemic Therapy Line 6**	**3 (0.4)**	**1 (0.4)**
Line 6: Anti-PD-1/PD-L1-based therapies	*1 (33.3)*	*0*
Cisplatin, irinotecan, pembrolizumab	1 (100)	0
Line 6: Anti-VEGF-based therapies	*1 (33.3)*	*0*
Bevacizumab-bvzr, paclitaxel protein-bound	1 (100)	0
Line 6: Single agent chemotherapy	*0*	*1 (100)*
Gemcitabine	0	1 (100)
Line 6: Other therapy	*1 (33.3)*	*0*
Dabrafenib, trametinib	1 (100)	0
**Systemic Therapy Line 7**	**1 (0.1)**	**0**
Line 7: Other therapy	*1 (100)*	*0*
Dabrafenib, hydroxyurea, trametinib	1 (100)	0

[1] Drug regimen classes are shown as a percentage of the relevant treatment line, with drug regimens shown as a percentage of the relevant regimen class. Mutually exclusive regimen classes were assigned in hierarchical order as shown, beginning with anti-PD-1/PD-L1-based therapy. Some agents in these next lines of therapy could be for treating NSCLC or another malignancy. Data are *n* (%), and percentages may not total 100 because of rounding.
PD1/PD-L1: programmed death 1/programmed death ligand-1; VEGF: vascular endothelial growth factor.

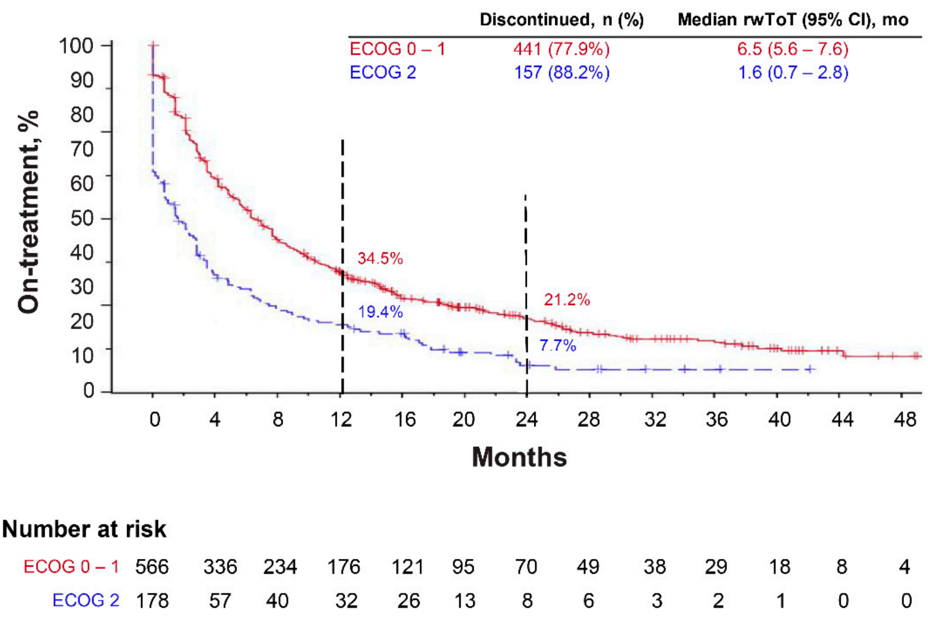

Figure S1. Kaplan–Meier plot depicting real-world time on treatment (rwToT) with pembrolizumab for patients with initial NSCLC diagnosis made at stage IV, according to ECOG performance status.
